# The ESF Programme on Integrated Approaches for Functional Genomics Workshop on ‘Proteomics: Focus on Protein Interactions’

**DOI:** 10.1002/cfg.109

**Published:** 2001-10

**Authors:** Joan Marsh

**Affiliations:** John Wiley & Sons, Ltd. Baffins Lane Chichester, West Sussex PO19 1UD UK


**Comparative and Functional Genomics**
*Comp Funct Genom* 2001; **2**: 319–326.
**DOI:** 10.1002/cfg.109

**Conference Report**

### The ESF programme on Integrated Approaches for Functional Genomics workshop on 'Proteomics: Focus on protein interactions'

11–13 May 2001, Villa Mondragone, Frascati, Rome, Italy

Joan Marsh, Publishing Editor
*John Wiley & Sons, Ltd., Baffins Lane, Chichester, West Sussex PO19 1UD, UK*

This inaugural meeting of the European Science Foundation's programme, 'Integrated Approaches for Functional Genomics', was opened by **Gianni Cesareni** (University of Rome Tor Vergata), one of the organisers. He compared proteomics to a jigsaw, but more difficult because it involves four dimensions and some of the pieces are still missing. Ideally, we would like to know the affinity of each protein for each of its binding partners. This will require much more work and there are several complementary approaches.

(i) Most current knowledge of protein-protein interactions resides in the published literature but it is not all readily accessible. Various groups are working on ways to extract this information and compile databases of protein-protein interactions (see Brannetti *et al.*, p. 314 and Blaschke *et al.*, p. 310).
(ii) Protein complexes can be isolated from cell extracts and the components identified.
(iii) There are experimental means to detect possible interactions among proteins, eg. yeast two-hybrid screening, but these do not necessarily reflect what happens inside the cell.
(iv) Characterisation of the specificity of individual protein-protein interactions.
(v) Protein interactions may be predicted computationally, based on structural information.

There are two views of the type of protein interactions that occur within a cell, which have different implications for cluster modelling. One sees a fixed complement of given complexes, with most proteins existing as multimers: this will give discrete unique clusters. The other sees many monomers in constant exchange among different multimeric complexes, which produces extended clusters.

### Genetic methods

The first formal presentation was given by **Pierre Legrain** (Hybrigenics, Paris) who discussed ways of comparing datasets from large-scale experiments searching for protein interactions (see Legrain, p. 310). Three important issues in this area are the incompleteness of these datasets, the abundance of false positive results, and the large size of the datasets, which result in the need for specific bioinformatic tools. He explained that the primary users of protein interaction data are biologists, who need exploratory tools, such as PIMRider$^{TM}$, developed by Hybrigenics. Other users of the data are bioinformaticians, who need the data to be deposited in a convenient, extractable format, allowing further analysis, such as clustering of interactions.

He also discussed the issue of regulated access: Hybrigenics provides PIMRider$^{TM}$ to academic laboratories for free, but because they are a commercial company they have to pay for, or may even be denied access to, many sources of data.

Dr Legrain's group uses the yeast two-hybrid system to detect protein interactions, whereas **Brian Kay** (University of Wisconsin) uses phage display of libraries of combinatorial peptides (see Kay, p. 304). He uses this approach to identify peptides that bind to a chosen target protein and then uses the consensus among the selected peptides to predict which protein might bind to the chosen protein in the cell.

As pointed out by Mike Taussig, this has the disadvantage of detecting only those interactions that involve short peptides and are independent of three-dimensional structure. Many of the

Copyright © 2001 John Wiley & Sons, Ltd.

interacting peptides are found to be on loops in protein structures, close to proline-rich regions that disrupt higher-order structure.

The final genetic approach to detecting protein interactions was described by **Andreas Plückthun** (University of Zurich) who exploits a Darwinian method of repeated diversification and selection for evolution of antibodies. This can be carried out *in vitro* for single selections using ribosome display. In this approach, the mRNA is completely translated but the nascent protein remains trapped in the ribosome complex together with the mRNA; the protein emerges through the ribosomal tunnel, whereupon the relevant domain can fold and bind to a target. The complex is stabilised by addition of $Mg^{++}$. Antibodies are the principal examples to date of proteins selected by ribosome display. For those that bind a given target, the mRNA is isolated and converted back into DNA by reverse transcription PCR under conditions that allow errors to be introduced (use of non proof-reading *Taq* polymerase); selection is then repeated, looking for proteins with improved binding affinity. This PCR-based system, which avoids bacterial transformation, can generate huge libraries very quickly; each cycle takes one day and libraries of $10^{11}$–$10^{12}$ proteins are produced. Greater numbers are possible, but Dr Plückthun said that it is preferable to perform further rounds of variation and selection than to increase the size of the library by 100-fold. The system is compatible with stringent selection conditions; many people consider ribosomes to be fragile components but the complexes are reasonably robust. This method has been used in conjunction with the synthetic Human Combinatorial Antibody Library (HuCAL) [1] to select antibodies against insulin and a DNA quadruplex structure. The combination of a synthetic library and secondary affinity maturation mimics the versatility of the immune response *in vitro.*

Selection conditions in ribosome display can be manipulated to favour certain properties amongst the newly evolved proteins. For example, for improved affinity, long off-rate selections (up to 250 hours) are used with biotinylated ligands in competition with an excess of unlabelled antigen; since the on-rate of most antibodies falls within a narrow range, off-rate selection alone produces antibodies with increased affinity.

Parallel screening of several antibodies and antigens simultaneously ('library versus library') can be achieved by moving the process inside the cell, and using growth selection to screen for interactions in a 'Protein Fragment Complementation Assay' (PCA). The idea is to cut an essential enzyme in half and allow the two parts to interact in the cell; reconstitution of the enzyme, in the correct geometry for activity, is mediated by a secondary interaction between an antigen and antibody. Thus, one half of the DNA encoding the enzyme, in this case dihydrofolate reductase (DHFR), is fused to the DNA for a single chain antibody, while the other half is fused to the DNA for a potential ligand. Binding of the antibody to ligand will reunite the halves, restoring enzyme activity and allowing the cells to grow on minimal medium. This system was shown to work in *Escherichia coli* and to possess the requisite specificity: when a mixture of antigen and antibody DNAs was used, such that 21 possible double transformants could be made, the only cells that grew were the three expressing cognate antigen-antibody pairs. This assay has the advantage of being fast, with simple handling; no expression or purification of antibody and antigen is necessary and only the DNA is needed. It also has a very high signal : noise ratio, with a $10^7$ fold difference between cognate and noncognate pairs. Its limitations are that both the antigen and the antibody must be stable in the cytoplasm (Dr Plückthun's group has constructed such an antibody library by removing disulphide bonds) and that the length of the linker needs to be optimised, which has also been done. The PCA suffers from the same restriction in library size as phage display, since it involves bacterial transformation, and the proteins must fold correctly in *E. coli*, which does not always happen. However, it will work with individual domains and is particularly sensitive, because only very small amounts of DHFR are required to restore growth. This assay does not select for high affinity binding, for which ribosome display is better, but does allow for the simultaneous isolation of several antigen-antibody interacting pairs.

### Peptide and protein chips

This session began with **Jens Schneider-Mergener** (Jerini AG, Berlin) describing the SPOT technology for highly parallel synthesis of peptides, in array format, on flat surfaces (see Schneider-Mergener, p. 307). These arrays can then be used for mapping protein-protein interactions, amongst other uses.

Copyright © 2001 John Wiley & Sons, Ltd. 

One application of this technique relies on selection, in this case using chemical mutagenesis to drive the evolution of new sequences.

The danger, pointed out by Gianni Cesareni, is that by testing all possible binding interactions amongst domains, one will find a ligand for any given target but if the natural ligand involves more than one binding motif, one may never find a true high affinity ligand. Brian Kay suggested synthesising a complete yeast or bacterial genome for use as a protein set: this has been considered but not yet attempted.

**Dolores Cahill** (Max-Planck-Institute of Molecular Genetics) has exploited the robotics developed by Hans Lehrach's group, originally for generation of high density DNA arrays, and has adapted these to high-throughput protein analysis. She described the strategy used to generate high-density arrays of thousands of proteins from a non-redundant cDNA library, where each protein is represented only once. This involves combining three well-known technologies, ie. cDNA library construction, recombinant protein expression and high density colony arraying. The proteins, which are His-tagged, are generally expressed from *E. coli*, but *Saccharomyces*, *Pichia pastoris* and *in vitro* expression are now also being explored. Robots pick bacterial colonies, array them into microplates and rearray the library according to selected coordinates onto PVDF membranes, where expression is induced.

Dr Cahill described work on a cDNA library prepared from human fetal brain mRNA (chosen because of the high number of genes expressed in this tissue compared with other tissues). After screening for in-frame protein expression using antibody to the His tag, and characterising clones using the hybridisation technique of oligonucleotide fingerprinting, a non-redundant UNIgene/UNIprotein set of about 15,000 clones was generated. Dr Cahill found an estimated 13,000 singly represented cDNA sequences, of which 60% represent full-length clones. Up to 4800 proteins could be arrayed on a standard microscope slide. Some of the drawbacks that Dr Cahill pointed out are that *E. coli* expression misses membrane proteins and proteins which are toxic to the bacteria, in addition, there are no glycosylations or post-translational modifications, and the proteins are denatured due to the conditions used to lyse the *E. coli*.

Applications of protein microarrays that Dr Cahill described include screening with antibodies, to identify the antigen ligand, and screening of patient sera. In one example, the protein array was screened with sera from autoimmune patients and positives were picked out, though the signals were sometimes weak and better results were obtained on the redundant set. When a positive is found, the protein can easily be identified, by sequencing the cDNA insert from the bacterial clone. The protein array is also being used for parallel selection of antibodies from phage display libraries, from which antibody arrays are being developed on glass or PVDF membranes. The aim for antibody arrays is to produce profiles of the proteins expressed in a given tissue. However, with an estimated 30–40,000 genes in the human genome, Dr Cahill believes that alternative splicing will produce about 10 times as many proteins: what fraction of these will be expressed in any given cell type, or detectable by the array, is not yet known.

**Ian Humphery-Smith** (University of Utrecht) is trying to produce antibody arrays that can be used to monitor the protein complement of all human tissues in an integrated approach to health and disease. The objective is to make a reporter matrix capable of following the protein output of every human gene, which will complement DNA array data on gene expression. The three reasons for going to an array-based approach to proteomics are parallelisation, miniaturisation and automation. Antibody arrays will allow screening of the entire human proteome, but the antibodies will first have to be produced and then screened against protein arrays. The traditional approach for assessing antibody cross-reactivity involves hybridisation against a panel of over 200 tissue sections, but most cell proteins are in low abundance, while most of the abundant proteins are common to all cells. To establish their specificity, antibodies must therefore be screened extensively on protein (antigen) arrays. The dilemma is to make antibody arrays relevant in time and cost of production. To try and obtain antibodies against every human protein, Dr Humphery-Smith is immunising mice in parallel with conserved exons, tagged with an immunogenic enhancer, which directs the antigen to antigen-presenting cells. In contrast to Andreas Plückthun, he believes that *in vitro* technology is not sufficient to replicate the full repertoire of the immune system. Because for every human gene there will be on average 10 products, the proteome is at least 400,000 proteins strong, and to produce antibodies against that number of antigens by any technology will be a daunting task; employing the conserved

Copyright © 2001 John Wiley & Sons, Ltd.

exon immunisation strategy enables one to follow gene product families. He believes that to get relevant antigenicity emulations in the form of protein arrays with which to screen the antibodies will take 2–3 years.

The real challenge is to produce antibody arrays cheaply, which means integrating robotics at each step in order to process thousands of antibodies against thousands of protein antigens. To do this, over the last two years Humphery-Smith's group has been developing prototype robotics designed to process more than 10 million ELISA equivalents every 2–3 hours. Optimal screening will require 100,000 different recombinant proteins on a chip, which will not be ready for 2–3 years, but the robot will be tested at 50,000 ELISA equivalents per day over the next six months. It is designed to screen 1500 different antibodies against 100,000 antigens in 100 million ELISA equivalents per day. These numbers are needed in order to get to grips with building affinity ligands against the elements on the human proteome. The objective in fact is to have five of these robots operating half a billion ELISA equivalents per day within the next three years in Utrecht.

The improvements in protein arrays that are needed are in the surface chemistry: arrays need a surface that will bind the antigen and nothing else, to avoid background and eliminate the need for blocking and washing. The antigen needs to be held in conditions that maintain its conformation: plenty of hydroxyl groups are used to mimic an aqueous environment. These conditions also need to be adjustable so that the binding of a given compound, be it a protein, nucleic acid or chemical ligand, to the protein array can be titrated. A problem with yeast two-hybrid assays is that many proteins will interact to a certain extent with some affinity. The protein-protein interaction studies now being performed will create vast amounts of data and Dr Humphery-Smith stressed the need for further investment in data management, processing and interrogation techniques.

Since the proteins of perhaps 10% of the genome cannot be expressed or even cloned, there is a need for a method to 'close' the genome to provide complete coverage. For this Humphery-Smith is identifying 'signature peptides', elements within proteins that have a functional or structural role in protein interactions; antibodies will be raised against these sequences as recombinant peptides and then screened for conformational cross-reactivity against protein arrays. Finally, he called for support for the newly-formed Human Proteome Organisation (**http://www.hupo.org/**).

### Mass spectrometry

The limitations of genome sequence for understanding biological function were beautifully illustrated by **Peter Roepstorff** (University of Southern Denmark, Odense), who asked us to consider a caterpillar and a butterfly: very different organism phenotypes but with the same genome sequence. Hence the need to study proteins and one of the most successful ways of doing this is by using mass spectrometry, which is central to proteomics and the qualitative and quantitative large scale analysis of complex protein mixtures. A second major challenge is to identify not just the core proteins, but also the post-translational modifications (PTMs), such as glycosylation, phosphorylation and acylation. As a result of these and other PTMs, current estimates are that there will be about 10 times as many molecular protein species as there are genes in a given organism.

Other issues to be addressed in proteomics include:

(i) Either eliminating the need for protein separation or finding an alternative method to 2D gels, which remain as much an art as a science. Multidimensional capillary separation is one possibility, but this has only a fraction of the separating power of current gels.
(ii) The poor quality of current genome annotation, which may be circumvented by searching crude genomic sequence using mass spectrometry data.
(iii) Quantitation: should this be performed at the separation stage, comparing spot intensities in gels, or during mass spectrometry?
(iv) Should protein interaction studies use gel-based or affinity-based techniques?

Dr Roepstorff termed detection of PTMs 'modification-specific proteomics' and introduced a new 'omics' - modificomics. He described detection of sites of phosphorylation using mass spectrometry on proteins isolated with anti-phosphotyrosine antibodies [3]. With MALDI mass mapping, alkaline phosphatase treatment directly on the target can be used to compare spectra to see where phosphate groups have been lost. He has successfully developed a means of detecting protein nitration, a

Copyright © 2001 John Wiley & Sons, Ltd.

modification which occurs in different infectious and stress conditions in the cell. In an exploratory model of *in vitro* nitration of BSA, Western blotting with an antibody against nitrotyrosine was used to loacte the nitrated protein. Nitrated peptides could be identified after in-gel digestion and the nitrotyrosines identified by electrospray ionisation mass spectrometry. This revealed that two peptides were nitrated, while precursor ion scanning for the immonium ion for nitrotyrosine revealed two additional partially nitrated peptides [4] He has also addressed the complexity of glycosylation, finding that $\gamma$-interferon possesses 13 putative glycan structures on Asn25 and 18 on Asn97 [6]. Such complicated glycosylation heterogeneity is probably important and a way nature uses to fine-tune interactions. Finally, in the area of protein interactions, Dr Roepstorff has used DNA attached to magnetic beads as a substrate to isolate DNA-binding proteins that were then characterised using MALDI mass spectrometry [2]. His philosophy is that the study of protein interactions should make use of all available tools in the hope that the results will all point in the same direction.

In response to questions, Dr Roepstorff explained that mass spectrometry can generate enough peptide sequence data to identify proteins whose gene sequence is known, while for proteins whose sequence is not in the databases, enough information can be obtained by MS/MS for cloning. For de novo sequencing, software programs are available to help with the interpretation of mass spectrometry data, although none provide automated sequencing from the mass spectra.

**Benedetta Mattei** (University of Rome, La Sapienza) presented studies combining surface plasmon resonance with mass spectrometry for the analysis of protein interactions, that she performed while working with Peter Roepstorff (see Mattei *et al.* next issue). In this approach, surface plasmon resonance is used to capture peptides interacting with a chosen protein, which are then identified by mass spectrometry. This can be used to map the domain of a protein that is forming the interaction surface.

A major development was described by **Carol Robinson** (University of Oxford) who has successfully applied quadrupole time-of-flight (Q-TOF) mass spectrometry to large complexes held together solely by non-covalent interactions. This is not a high-throughput approach but the elegance of the experiments impressed everyone present. She uses nanoflow electrospray with low volumes in very small capillaries and allows evaporation to proceed slowly. A gentle pressure gradient guides the intact complex across the chamber. Slightly increasing the amount of energy in the system causes the complex to break up into its more stable constituents, providing valuable information about the probable routes of assembly. Dr Robinson studied a recently described molecular chaperone, MtGimC, which was shown to consist of two $\alpha$ and four $\beta$ subunits. Further examination showed that the assembly process was highly cooperative, with no intermediates being detected.

Dr Robinson has also investigated protein-RNA interactions, which are stronger than protein-protein interactions in the gas phase and thus rather more difficult to study. TRAP is an RNA-binding protein that was known to bind tryptophan. She showed that in the absence of tryptophan, 11 TRAP monomers associate autonomously: addition of tryptophan led to cooperative binding. She also confirmed the existence of a 22-mer, which had been postulated but never proven.

Another experiment looked at the composition of the *E. coli* ribosome, a huge complex of three RNA molecules and 55 different proteins. This can be maintained intact in the mass spectrometer, but lowering the $Mg^{++}$ concentration causes it to dissociate into the 50S and 30S subunits. Further controlled dissociation of the 50S particle led to the identification of pentamers and hexamers, as well as monomers, elucidating the structure of the ribosome [5]. This paves the way for dynamic studies of the ribosome's response to therapeutic agents. Finally, Dr Robinson mentioned studies of the yeast spliceosome. This RNA-protein complex has never been crystallised. By dissecting the subunit composition, she has obtained useful information on the stability of the various components, which should facilitate crystallisation attempts.

**Giulio Superti-Furga** (CellZome GmbH, Heidelberg) has focused on protein complexes in a different way, exploiting the fact that tyrosine phosphorylation is almost invariably associated with protein-protein interactions. Endogenous genes are tagged *in situ* then translated. Phosphatase activity is blocked using vanadate, then the proteins are immunoprecipitated with an anti-phosphotyrosine antibody. The proteins are purified using tandem affinity purification, which is reliable and robust and can be adapted to high-throughput studies: the system is now handling 4000 MALDI samples per

Copyright © 2001 John Wiley & Sons, Ltd.

week. The aim is to identify all the protein complexes in *Saccharomyces cerevisiae*. The data are automatically compared with binary interactions listed in the Yeast Protein Database; novel components are then annotated manually. This is an ambitious project that had just come on-stream at the time of the workshop but which should soon be providing useful information. There are limitations, as raised in the discussion: for example, this method will not detect membrane proteins, nor does it say anything about the stoichiometry or the specificity of the interactions. Pierre Legrain pointed out that researchers see protein interactions in different ways: geneticists are interested in pathways, linking proteins in a linear chain of function, whereas biochemists are interested in complexes that associate to perform a given task. He also raised the issue of subcellular localisation: many proteins are active only within a given cellular compartment and any interactions detected outside of that compartment probably bear little relevance to their function. Dr Superti-Furga acknowledged these caveats but emphasised that collection of the data is just the beginning, interpretation of the data will be the real challenge.

### Bioinformatics

The first session was concerned with the annotation of genomic and protein databases. **Michael Sternberg** (Imperial College, London) is exploiting the rapidly increasing repository of information on protein structure to understand more about structure-function relationships. Integrase and ribonuclease H show very little sequence homology but have conserved structural homology, particularly around the active site. Chymotrypsin and subtilisin perform the same function but use different protein folds to do so. On the other hand, lysozyme and $\alpha$-lactalbumin share almost identical structures but one is enzymatically active and the other is not.

Automated methods for protein structure prediction are gradually improving, and homology modelling has revealed that most amino acid changes occur in loops and exposed areas of proteins. When two proteins show good structural homology, an automated prediction is nearly as accurate as a manual one. However, when the homology falls below 40%, manual input becomes essential.

In Dr Sternberg's automated analysis, the program 3D-JIGSAW (**http://www.bmm.icnet.uk/servers/3djigsaw/**) compares the sequence of a query protein to the templates in their library. If there is no direct structural 'hit', then a fold recognition algorithm, 3D-PSSM (**http://www.bmm.icnet.uk/~3dpssm/**), is applied to model the fold using known structural information, this can detect more remote homologies that PSI-BLAST misses. Dr Sternberg then takes information on biological function derived from text searches of the PubMed archive and uses this to manually rank the target proteins proposed by 3D-PSSM. Last year, this technique proved the most efficient in a CASP4 open competition for determining structures that excluded novel folds. It could be used to facilitate future drug development, specifically in the design of antagonists or inhibitors to fit into active site pockets.

**Thure Etzold** (European Bioinformatics Institute) chose to focus on the problems raised by the explosion in the amount of protein interaction data and the need for an integrated solution. There are many collections of protein interaction data being assembled and many use their own set of standards, which in turn are based on different assumptions. The NCBI developed standards for its own use that have since been adopted more widely; ACeDB was developed to store genome data for the worm *Caenorhabditis elegans* and was subsequently expanded for more general use; Array Express is not a standard, but is a technology that has found broad application.

Dr Etzold classified people who build solutions as generalists or pragmatists: the former focus on flexible and extensible solutions that are often slow and do not match the detailed requirements of a particular task; the latter create a system that does exactly what is required initially but cannot be readily adapted and is often short lived. Lion Biosciences' solution was to create a network of databases, the SRS library network, which has explicit external references, such as a link to a specific database, and implicit cross-references, in the form of common fields and standard terms within those fields, for example, correct taxonomical terms for organisms and Gene Ontology nomenclature for genes and their functions. The problem with this approach is that there are many sources of error: errors in the databases, changes to content or format, and differences in concept. The envisaged solution is a database that has frequent, automatic and extensive consistency checks. Such an integrated database would need to enable scientists to create data viewers, see analysis flows,

Copyright © 2001 John Wiley & Sons, Ltd.

add or change the integrated databanks and the links between them. All of this would need to be integrated with analysis tools. SRS has a descriptive language for such extensions but users may perceive this as additional programming, therefore it requires a graphical interface.

Peter Roepstorff asked whether, as an extreme example, a PhD student should be encouraged to develop a new database or persuaded to use the existing technology to avoid further confusion. Thure Etzold replied that new technology is always needed as the field matures and creativity should be encouraged. As standard tools are more widely adopted, it becomes easier for new features to be integrated into existing structures. Pierre Legrain called for a more systematic gene nomenclature: the current system has evolved over 100 years with no clear pattern so that many gene names do not refer to the primary function of the gene product. He said that if biologists do not tackle this problem now, it will be even worse later. Thure Etzold replied that the Gene Ontology scheme is making progress in this area.

The second half of the Bioinformatics session addressed the issue of trying to extract information on protein interactions from the published literature. **Manuela Helmer Citterich** (University of Rome, Tor Vergata) described iSPOT, a web tool for inferring protein-protein interactions (see Brannetti *et al.*, p. 314), and MINT, a database dedicated to protein interactions (see Wixon, p. 338). iSPOT does not need the 3D structure of the domains under study and can make a prediction as long as their sequences can be confidently aligned to domains of known structure from the same families. The reliability of the prediction depends on the level of sequence identity between the query domains and the domains whose experimentally determined binding data have been used to train the software.

**Rita Casadio** (University of Bologna) presented a method for the prediction of protein-protein interaction sites, based on neural networks. The aim is to identify those regions on a protein surface that interact with other proteins. She used information from several databases amounting to several thousand known interactions among over 4000 proteins to look at shape, chemical complementarity, and the distribution of charged and polar residues but found no characteristics that typified protein-protein interacting surfaces. Such surfaces showed the same residue composition as other regions of the proteins.

Dr Casadio examined bacterial luciferase, which comprises a heterodimer that has been crystallised. She conceptually dissected the crystal and studied the interacting surfaces. This process was repeated for many known examples and the information was used to 'train' a neural network to extract general rules concerning protein interactions. These were stored in an algorithm that could, when given information about a new structure, predict which parts of the protein were most likely to form the interface. For a mouse antibody fragment, the algorithm correctly detected 73% of interacting residues; although it did miss some and gave a couple of false positive results. This method could be useful in complementing results from other studies in proteomics. However, one drawback in using data from individual protein structures is that there may be conformational changes during complex formation.

**Jong Park** (MRC-Dunn Institute, Cambridge) believes that the direct interaction space for proteins is too big (with many millions of possible interactions) for it to be possible to globally map them. He is taking a different approach, constructing a general protein interaction map using structural information from the Protein Data Bank (PDB). By applying a strict criterion of structural interaction, he is able to construct a broad and general, but definite, map. He defines a domain arbitrarily, merging structural and sequence alignment data. He then incorporates phylogenetic information on protein family interactions, and data on fold detection and classification. These datasets are then used to predict interactions among homologues, but the data have to be validated. The outcome is PSIMAP (**http://www.biointeraction.net/**), which contains information on all known domains. Dr Park proposes that intra- and intermolecular interactions are fundamentally different. He states that protein folds have diverse and distinct repertoires of interactions, which should ultimately make their classification simpler, but there is still a long way to go.

In response to questions, Dr Park conceded that this method cannot deal with protein interactions that are mediated by unstructured regions and that he has not yet examined why interactions between certain pairs of folds are favoured. Andreas Pluckthun pointed out that the immunoglobulin domain, while stable, is used differently in various receptors: cellular receptors present the edge of the domain for binding, whereas antibodies use a more central part of the domain. Dr Park replied that

Copyright © 2001 John Wiley & Sons, Ltd.

immunoglobulins were a special case, but he felt that his method could map the majority of interactions that occur in a general way.

The meeting was closed by **Alfonso Valencia** (Protein Design Group, Cantoblanco, Madrid) who described his work on extracting information concerning protein interactions from the published literature (see Blaschke *et al.*, p. 310) and a method for prediction of protein interactions. The prediction method looks for correlated mutations between possible interaction partners and also for complementarity of the phylogenetic trees of potentially interacting proteins, which could indicate co-evolution of the proteins.

### References

1. Knappik A, Ge L, Honegger A, *et al.* Fully synthetic human combinatorial antibody libraries (HuCAL) based on modular consensus frameworks and CDRs randomized with trinucleotides. *J Mol Biol* 2000; **296**(1): 57–86.
2. Nordhoff E, Krogsdam AM, Jorgensen HF, *et al.* Rapid identification of DNA-binding proteins by mass spectrometry. *Nat Biotechnol* 1999; **17**(9): 884–888.
3. Pandey A, Podtelejnikov AV, Blagoev B, Bustelo XR, Mann M, Lodish HF. Analysis of receptor signalling pathways by mass spectrometry: Identification of Vav-2 as a substrate of the epidermal and platelet-derived growth factor receptor. *Proc Natl Acad Sci USA* 2000; **97**: 179–184.
4. Petersson AS, Steen H, Kalume DE, *et al.* Investigation of tyrosine nitration in proteins by mass spectrometry. *J Mass Spectrom* 2001; **36**(6): 616–625.
5. Rostom AA, Fucini P, Benjamin DR, *et al.* Detection and selective dissociation of intact ribosomes in a mass spectrometer. *Proc Natl Acad Sci U S A* 2000; **97**(10): 5185–5190.
6. Sareneva T, Mortz E, Tolo H, Roepstorff P, Julkunen I. Biosynthesis and N-glycosylation of human interferon-gamma. Asn25 and Asn97 differ markedly in how efficiently they are glycosylated and in their oligosaccharide composition. *Eur J Biochem* 1996; **242**: 191–200.

---

This Conference Report aims to present a commentary on the topical issues in genomics studies presented at the conference. The article represents a personal critical analysis of the reports made at the meeting and aims at providing implications for future genomics studies. This Conference Report was written by Joan Marsh - Publishing Editor, John Wiley and Sons, Ltd, with assistance from Michael Taussig - Chairman of the ESF programme on Integrated Approaches for Functional Genomics.

Copyright © 2001 John Wiley & Sons, Ltd. 
